# Supplementary material for: Generation of an isoform-level transcriptome atlas of macrophage activation
Source: J Biol Chem. 2021 May 14;296:100784. doi: 10.1016/j.jbc.2021.100784 (PMC8191339; doi:10.1016/j.jbc.2021.100784)
Supplement: Supplemental Figures S1 and S2 [file mmc2.pdf]

Supplemental Information for

Isoform-level transcriptome Atlas of Macrophage Activation

by

Apple Cortez Vollmers, Honey Mekonen, Sophia Campos, Susan Carpenter, Christopher Vollmers

Content:

Figure S1: Read characteristics of different R2C2 libraries
Figure S2: RT-qPCR validation of alternative exon usage

=-

Supplemental tables S1-9 are supplied separately as a single .xls file.

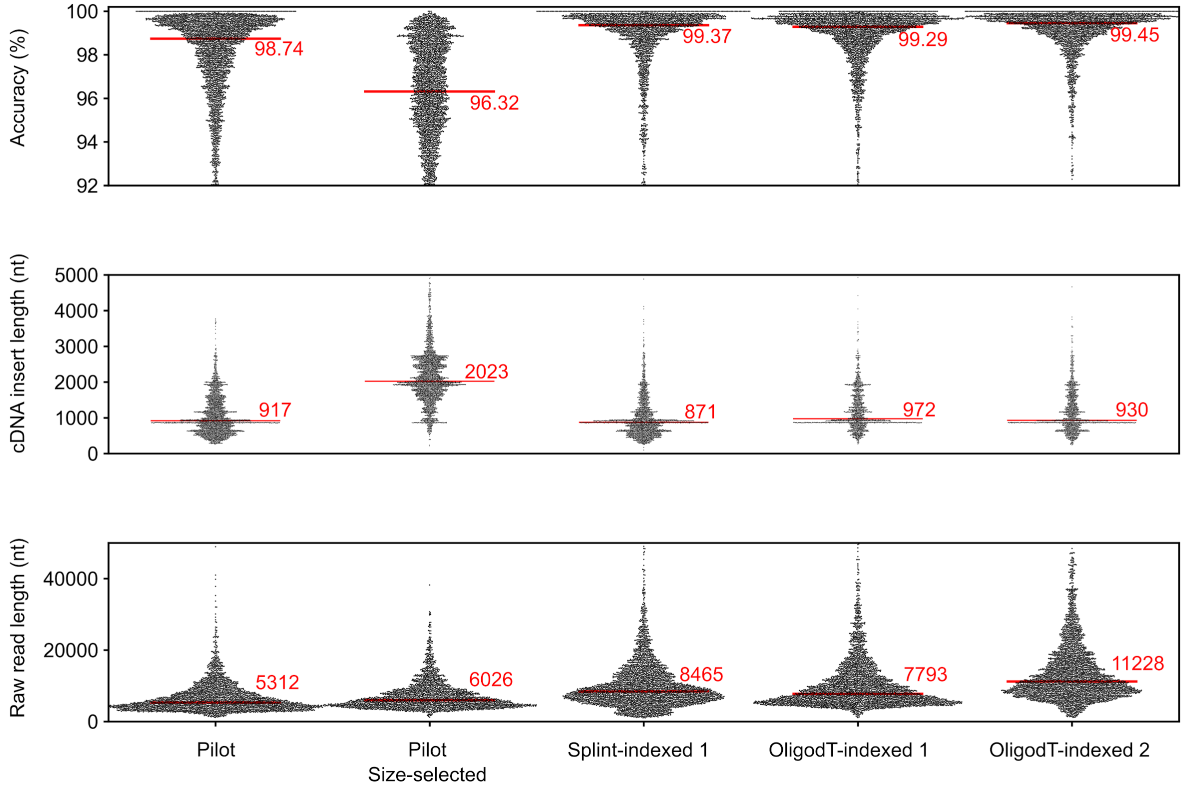

Fig. S1: Read characteristics of different R2C2 libraries. Accuracy, cDNA insert length, and ONT raw read length of individual R2C2 reads are shown for different R2C2 libraries as swarmplots, with median values indicated by red lines and numeric values. Splint-indexed 1, OligodT-indexed 1, and OligodT indexed 2 libraries were size-selected by gel excision with OligodT-indexed 2 undergoing the most stringent size-selection.

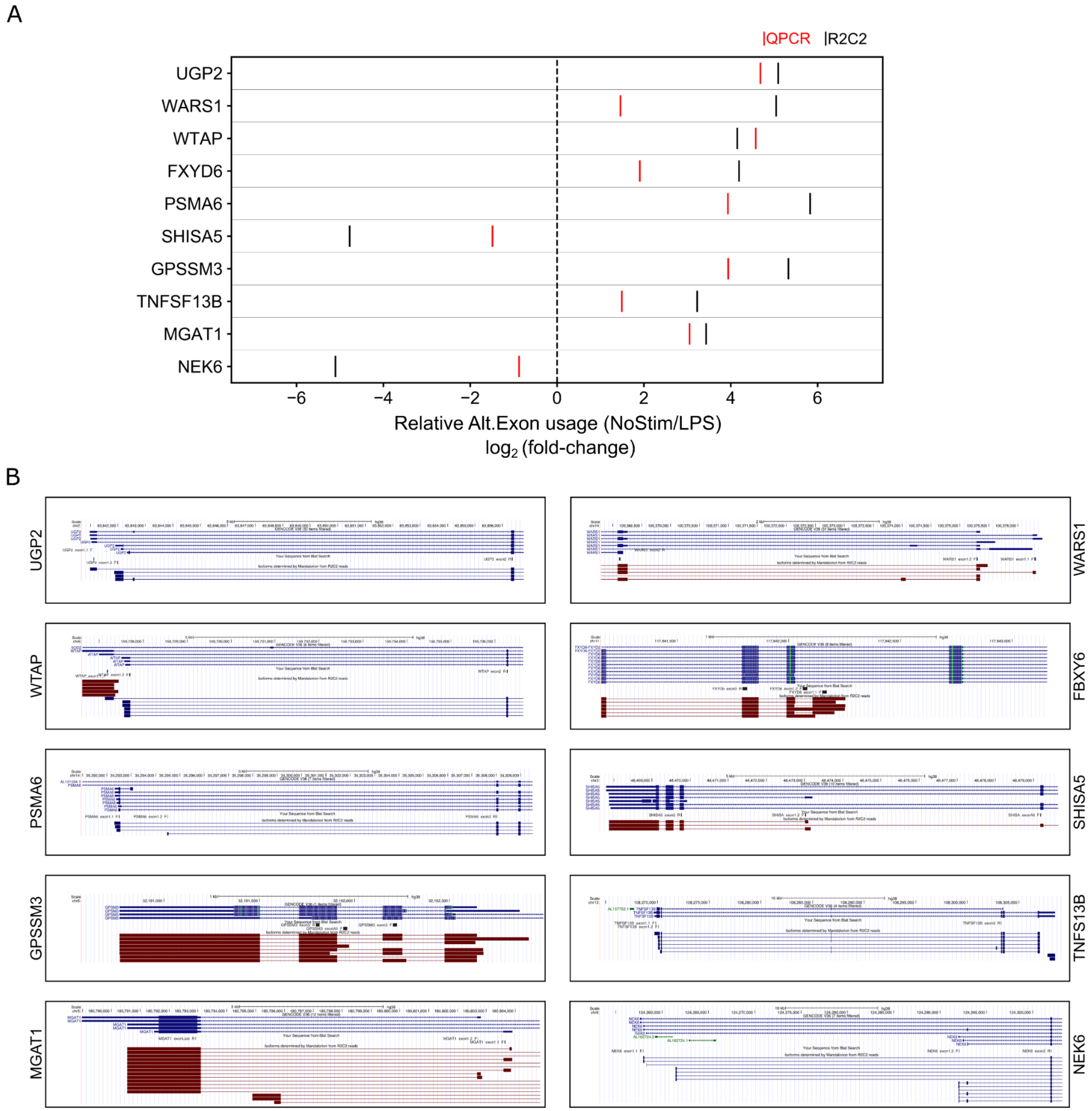

Fig. S2: RT-qPCR validation of alternative exon usage. A) The fold-change between NoStim and LPS conditions of relative alternative exon usage is shown for 10 genes. Values were determined by RT-qPCR for a third donor (red) and compared to values determined by R2C2 for the other two donors in the study. Relative alternative exon usage was defined as “exon” expression/ ”alt. exon” expression, with expression determined as delta(Ct) (RT-qPCR) or read numbers (R2C2) (Table S7). Fold-change was determined by calculating relative alternative exon usage (NoStim) / relative alternative exon usage (LPS). In this way, potential biases introduced through primer design and amplification efficacy are minimized. B) The position of the primers used for RT-qPCR are shown as UCSC Genome Browser shots. Primers were designed so primers designed for the alternative exons shared a reverse primer designed for a shared exon.
